# Activation of Peroxisome Proliferator-Activated Receptor Gamma by Rosiglitazone Increases Sirt6 Expression and Ameliorates Hepatic Steatosis in Rats

**DOI:** 10.1371/journal.pone.0017057

**Published:** 2011-02-23

**Authors:** Soo Jin Yang, Jung Mook Choi, Seoung Wan Chae, Won Jun Kim, Se Eun Park, Eun Jung Rhee, Won Young Lee, Ki Won Oh, Sung Woo Park, Sun Woo Kim, Cheol-Young Park

**Affiliations:** 1 Diabetes Research Institute, Sungkyunkwan University School of Medicine, Kangbuk Samsung Hospital, Seoul, Korea; 2 Department of Pathology, Sungkyunkwan University School of Medicine, Kangbuk Samsung Hospital, Seoul, Korea; 3 Department of Endocrinology and Metabolism, Sungkyunkwan University School of Medicine, Kangbuk Samsung Hospital, Seoul, Korea; Mayo Clinic, United States of America

## Abstract

**Background:**

Sirt6 has been implicated in the regulation of hepatic lipid metabolism and the development of hepatic steatosis. The aim of this study was to address the potential role of Sirt6 in the protective effects of rosiglitazone (RGZ) on hepatic steatosis.

**Methods:**

To investigate the effect of RGZ on hepatic steatosis, rats were treated with RGZ (4 mg·kg^−1^·day^−1^) by stomach gavage for 6 weeks. The involvement of Sirt6 in the RGZ's regulation was evaluated by Sirt6 knockdown in AML12 mouse hepatocytes.

**Results:**

RGZ treatment ameliorated hepatic lipid accumulation and increased expression of Sirt6, peroxisome proliferator-activated receptor gamma coactivtor-1-α (Ppargc1a/PGC1-α) and Forkhead box O1 (Foxo1) in rat livers. AMP-activated protein kinase (AMPK) phosphorylation was also increased by RGZ, accompanied by alterations in phosphorylation of LKB1. Interestingly, in free fatty acid-treated cells, Sirt6 knockdown increased hepatocyte lipid accumulation measured as increased triglyceride contents (p = 0.035), suggesting that Sirt6 may be beneficial in reducing hepatic fat accumulation. In addition, Sirt6 knockdown abolished the effects of RGZ on hepatocyte fat accumulation, mRNA and protein expression of Ppargc1a/PGC1-α and Foxo1, and phosphorylation levels of LKB1 and AMPK, suggesting that Sirt6 is involved in RGZ-mediated metabolic effects.

**Conclusion:**

Our results demonstrate that RGZ significantly decreased hepatic lipid accumulation, and that this process appeared to be mediated by the activation of the Sirt6-AMPK pathway. We propose Sirt6 as a possible therapeutic target for hepatic steatosis.

## Introduction

Hepatic steatosis is a relatively benign and early condition of nonalcoholic fatty liver disease (NAFLD), which is strongly associated with obesity and insulin resistance [1], [2]. But, when accompanied by other metabolic disorders, it can progress to severe stages of NAFLD; steatohepatitis, cirrhosis and hepatocellular carcinoma [3]–[5].

Thiazolidinediones (TZD) are agonists of peroxisome proliferator-activated receptor gamma (PPARγ), a nuclear transcription factor primarily involved in insulin action, lipid and glucose metabolism, and energy homeostasis [6], [7]. TZD-activated PPARγ improves insulin sensitivity in most tissues, but affects lipid metabolism in a cell- and tissue-specific manner. In adipose tissue, TZD-activated PPARγ stimulates adipocyte differentiation and induces lipogenic enzymes, thereby increasing fat storage, especially into subcutaneous adipose tissue [8]–[10]. This sequestration of lipid into adipose tissue decreases circulating levels of triglyceride (TG) and free fatty acids (FFA), thus decreasing lipid uptake in the liver. In addition, TZD stimulates fatty acid oxidation and inhibits excessive lipolysis and fatty acid synthesis in the liver, resulting in reduced hepatic fat contents in both rodents and humans [11]–[13]. Although the link between TZD and hepatic steatosis has become more evident, the underlying mechanism is poorly understood. Several lines of evidence suggest that TZD activates AMP-activated protein kinase (AMPK) by both PPARγ-dependent and -independent pathways, implying that AMPK may be a key regulator of the beneficial metabolic effects of TZD [14]–[17].

Sirtuins are a family of proteins with NAD^+^-dependent deacetylase and ADP-ribosyltransferase activities. They control key cellular and physiological processes including apoptosis, energy homeostasis, mitochondrial function and longevity [18]. Seven mammalian sirtuins (Sirt1-7) have been characterized according to their distinct localization patterns and biological functions [18], [19], and have been suggested as potential therapeutic targets for metabolic syndrome [20]. Sirt1 is a well-studied nuclear sirtuin with beneficial metabolic effects, and it has been implicated in the improvement of hepatic steatosis [21], [22]. Sirt1 overexpression in high-fat diet-fed mice results in improved glucose tolerance and protection from hepatic steatosis and inflammation compared with wild-type controls [22]. Several lines of evidence suggest that Sirt1 may function in part by activating AMPK [23], [24], which is one of the suggested mechanisms underlying TZD's beneficial effects. Similarly, Sirt6 is also localized in the nucleus and may also be involved in metabolic regulation. Sirt6-deficient mice have an aging phenotype and metabolic defects in glucose homeostasis, and eventually die at about four weeks of age [25]. Interestingly, transgenic mice overexpressing Sirt6 are protected from hepatic fat accumulation and other metabolic damages induced by a high-fat diet [26]. Considering the previous findings implicating AMPK in TZD's beneficial effects, and the potential interdependence of AMPK and sirtuins, we speculated that TZD may protect against hepatic steatosis by activating the Sirt6-AMPK pathway.

This study was designed to test whether Sirt6 contributes to the protective effects of the TZD rosiglitazone (RGZ) on hepatic steatosis. Using a rat model of moderate obesity and insulin resistance and a cell model of hepatocyte steatosis, we report up-regulation of adiponectin, Sirt6, peroxisome proliferator-activated receptor gamma coactivtor-1-α (Ppargc1a/PGC1-α) and forkhead box O1 (Foxo1), as well as increases in both LKB1 and AMPK activities upon RGZ treatment. The functional relevance of Sirt6-stimulated AMPK activation in the RGZ's regulation on hepatic fat accumulation was further investigated by RNA interference (RNAi)-mediated Sirt6 knockdown in AML12 mouse hepatocytes. Sirt6 knockdown abolished the effects of RGZ on hepatocyte fat accumulation and the Sirt6-AMPK pathway, suggesting that Sirt6 is involved in RGZ-mediated metabolic effects. Collectively, these data demonstrate that attenuation of hepatic steatosis by RGZ is likely mediated by the activation of the Sirt6-AMPK pathway, suggesting Sirt6 as a therapeutic target for hepatic steatosis and its related diseases.

## Methods

### Animals

Male Otsuka Long-Evans Tokushima Fatty (OLETF) rats and age-matched Long-Evans Tokushima Otsuka (LETO) rats were provided by Otsuka Pharmaceutical (Tokushima, Japan). Rats were maintained in a temperature- and humidity-controlled room with a 12 h light/dark cycle and fed PicoLab Rodent Diet 20 5053 (5% wt/wt fat; Purina Mills, Richmond, IN, USA) with unlimited access to food and water. To investigate the effect of RGZ on hepatic steatosis, total 18 rats at about 32 weeks of age were treated with RGZ (4 mg·kg^−1^·day^−1^; Cayman, Ann Arbor, MI, USA) or with PBS as control vehicle, via stomach gavage for 6 weeks. The study protocol conformed to the specifications outlined in the National Institutes of Health's Guiding Principles for the Care and Use of Laboratory Animals and was approved by the Institutional Animal Care and Use Committee of the Sungkyunkwan University Kangbuk Samsung Hospital (Approval ID: 201010013).

### Blood and Tissue Collection

Thirty eight-week-old LETO and OLETF rats were anesthetized with intraperitoneal Zoletil/Rompun after an overnight fast, and all efforts were made to minimize suffering. Blood was collected from the abdominal aorta. After blood collection, tissues were harvested and stored at −80°C until further analysis.

### Cells and Culture Conditions

AML12 mouse hepatocytes (American Type Culture Collection, Manassas, VA, USA) were cultured in DMEM/F-12 media (Invitrogen, Carlsbad, CA, USA) supplemented with 10% FBS, antibiotics (100 units/ml penicillin and 100 µg/ml streptomycin), 0.1 µM dexamethasone, and a mixture of insulin, transferrin, and selenium (Invitrogen). For the induction of hepatocyte steatosis, subsets of AML12 hepatocytes were treated with palmitic acid (250 µM; Sigma-Aldrich, St Louis, MO, USA; FFA) for 48 h. This was followed by treatment with FFA and/or RGZ (10 µM) for an additional 24 h.

RNAi-mediated gene silencing was performed according to the manufacturer's instructions. AML12 cells were transfected with negative control siRNA (Stealth RNAi negative control duplexes; Invitrogen) or Stealth RNAi siRNA targeting Sirt6 using Lipofectamine (Invitrogen). After 24 h of transfection, subsets of cells were incubated with FFA for 48 h to induce hepatocyte steatosis. Cells were then cultured in the presence or absence of FFA and/or RGZ for an additional 24 h.

### Histological Analysis

Livers were fixed overnight in 10% (vol./vol.) zinc formalin, dehydrated in a graded series of alcohol washes, cleared in toluene and embedded in paraffin. Using a microtome, 5 µm sections were generated, collected on slides, and then stained with hematoxylin and eosin.

### NAFLD Activity Score (NAS)

The NAS is used to assess the severity of NAFLD, according to Kleiner et al. [27]. The NAS evaluates three features of NAFLD and is calculated by adding the individual scores: steatosis (<5% = 0, 5–33% = 1, 33–66% = 2, >66% = 3), lobular inflammation (none  = 0, <2 foci  = 1, 2–4 foci  = 2, >4 foci  = 3), and ballooning (none  = 0, few  = 1, many  = 2). Liver sections were evaluated for NAS by a pathologist blinded to the study.

### Metabolic Parameters

Glucose, TG and glycerol concentrations were measured by enzymatic assays (Sigma). FFA concentrations in serum, tissues, and cell extracts were measured by a commercial kit from Wako (Wako Pure Chemical Industries, Osaka, Japan). Total cholesterol (TC) concentrations were measured by a cholesterol assay kit (Cayman). All values of TG, glycerol, FFA, and TC in liver and cell extracts were normalized to protein concentrations. Commercially available ELISA kits were used for the measurement of adiponectin (B-Bridge International, Mountain View, CA, USA) and insulin (Crystal Chem, Downers Grove, IL, USA).

### Isolation of Total RNA and Quantitative RT-PCR

Total RNA was isolated from cells and tissues with the use of a PureLink RNA Mini Kit (Invitrogen). Reverse transcription was performed using a High-Capacity RNA-to-cDNA kit (Applied Biosystems, Foster City, CA, USA) following the manufacturer's instructions. mRNA expression was quantified by real-time PCR (LightCycler 480 system; Roche, Indianapolis, IN, USA). Synthesized cDNA was mixed with LightCycler 480 Probes Master Mix (Roche), and with a gene-specific primer and probe mixture (Universal ProbeLibrary system; UPL; Roche; Table 1). Individual reactions for target and β-actin (*Actb*) or glyceraldehyde-3-phosphate dehydrogenase (*Gapdh*) were carried out separately with negative controls lacking cDNA. The conditions used were as follows: 95°C for 10 min, followed by 40 cycles of denaturation (95°C for 10 s) and annealing/extension (60°C for 20 s). The cycle number for the threshold of detection was determined by LightCycler 480 software (Roche). mRNA expression of each target was normalized to that of the *Actb* or *Gapdh* gene and expressed as fold change relative to controls.

**Table 1:** Primers sequences used in real-time RT-PCR.

Name of primers	Forward	Reverse	UPL No.
mAdipoq	ggagagaaaggagatgcaggt	ctttcctgccaggggttc	17
mPpara	ctgagaccctcggggaac	aaacgtcagttcacagggaag	91
mPparg	gaaagacaacggacaaatcacc	gggggtgatatgtttgaacttg	7
mSirt1	tcgtggagacatttttaatcagg	gcttcatgatggcaagtgg	104
mSirt6	acgcggataagggcaagt	ctcccacaccttgcgttc	82
mPpargc1a	gaaagggccaaacagagaga	gtaaatcacacggcgctctt	29
mFoxo1	cttcaaggataagggcgaca	gacagattgtggcgaattga	11
mActb	ggatgcagaaggagattactgc	ccaccgatccacacagagta	63
rAdipoq	tggtcacaatgggataccg	cccttaggaccaagaacacct	80
rPpara	tgcggactaccagtacttaggg	gctggagagagggtgtctgt	116
rPparg	ggtgaaactctgggagatcct	aatggcatctctgtgtcaacc	115
rSirt6	acctaacgctcgctgatga	gaggtacccagggtgacaga	18
rPpargc1a	aatttttcaagtctaactatgcagacc	aaaatccagagagtcatacttgctc	38
rFoxo1	tcaggctaggagttagtgagca	ggggtgaagggcatcttt	68
rGapdh	gagatcaacgtgttccagtgc	cttccaccacgtagggattc	1

mActb, mouse β-actin; mAdipoq, mouse adiponectin; mFoxo1, mouse forkhead box O1; mPpara, mouse peroxisome proliferator-activated receptor alpha; mPparg, mouse peroxisome proliferator-activated receptor gamma; mPpargc1a, mouse peroxisome proliferator-activated receptor gamma coactivtor-1-α; rGapdh, rat glyceraldehyde-3-phosphate dehydrogenase; UPL, Universal ProbeLibrary system.

### Western Blot Analysis

Total protein was isolated from tissues and cells by homogenization in cold RIPA lysis buffer (Amresco, Solon, OH, USA) containing protease inhibitors and phosphatase inhibitors (Roche). The lysates were centrifuged, and supernatants were collected and subjected to western blot analysis. Protein concentrations were measured using the Bio-Rad protein assay (Bio-Rad Laboratories, Hercules, CA, USA) according to the manufacturer's instructions. Western blotting was performed by denaturing 50 µg of protein at 95°C for 5 min in Laemmli sample buffer (Fermentas, Burlington, Ontario, Canada). Sample proteins were separated by SDS-PAGE and transferred to a polyvinylidene difluoride membrane. Membranes were blocked in 5% nonfat dry milk in Tris-buffered saline/Tween-20 (50 mM Tris, pH 7.5, 500 mM sodium chloride, and 0.05% Tween-20) for 1 h at room temperature. Membranes were incubated overnight at 4°C with primary antibodies for Foxo1, insulin receptor (IR), IR substrate1 (IRS1), Akt, phospho-Akt (Ser473), AMPKα, phospho-AMPKα (Thr172), LBK1, phospho-LKB1 (Ser428) (Cell Signaling Technology, Danvers, MA, USA), PGC1-α, β-actin (Santa Cruz Biotechnology, Santa Cruz, CA, USA), phospho-Foxo1 (Ser249) (Invitrogen), Sirt6, phospho-IR (Tyr1158, Tyr1162, and Tyr1163), phospho-IRS1 (Tyr612), and glucose transporter 1 (GLUT1) (Abcam, Cambridge, UK). Membranes were then exposed to an anti-rabbit secondary antibody conjugated to horseradish peroxidase (Santa Cruz Biotechnology) for 1 h at room temperature. Signals were detected by chemiluminescence using the ECL detection reagent (GE Healthcare, Piscataway, NJ, USA). The bands were scanned by a Geliance 600 Imaging System (PerkinElmer, Waltham, MA, USA) with a cooled 12-bit camera, and quantified by densitometry. Levels of phosphorylated Foxo1 at Ser249, AMPKα (Thr172), LKB1 (Ser428), IR at three tyrosine residues (Tyr1158, Tyr1162, and Tyr1163), IRS1 at Tyr612, and Akt at Ser473, were normalized to values for Foxo1, AMPKα, LKB1, IR, IRS1, and Akt, respectively. Levels of Sirt6, PGC1-α, and GLUT1 were normalized to values for β-actin.

### Statistical Analysis

All statistical analyses were performed using PASW Statistics 17 (SPSS Inc., Chicago, IL, USA). Data are expressed as means±SEM. Student's *t* test or one-way ANOVA was performed to compare groups. Statistical significance was defined as p<0.05.

## Results

### RGZ Improved Hepatic Steatosis in OLETF Rats

To address the role of Sirt6 in the beneficial effects of RGZ, we first determined the metabolic effects of RGZ on hepatic steatosis. After 6 weeks of treatment, it is noticeable that body weight of OLETF control rats decreased, which is due to aggravation of diabetic conditions (Table 2). However, there was a significant increase in the body weight of RGZ-treated OLETF rats without altered food intake (Table 2). Total fat weights of RGZ-treated OLETF rats were significantly higher than those of control OLETF rats (Table 2), consistent with previous findings that RGZ leads to increased fat storage into adipose tissue [8]–[10]. There was a significant increase in subcutaneous fat and mesenteric fat in LETO rats, and in subcutaneous fat and retroperitoneal fat in OLETF rats with RGZ treatment, but the patterns of increases in the other fat pads were not statistically significant.

**Table 2:** The effects of rosiglitazone (RGZ) on body weights, food intake and fat pad weights.

	LETO		OLETF	
	Control	RGZ	Control	RGZ
Body weight				
Baseline (g)	511±14	515±9	603±23	611±21
Post-treatment (g)	522±8	559±17	530±15	635±43
Weight change (g)	12±11	45±16	−73±10	25±30*
Food intake (g)	23.1±0.5	23.4±2.3	39.2±0.4	37.9±0.6
Fat pad weights (%)	3.9±0.4	4.8±0.4	5.7±0.8	11.3±1.2*
Subcutaneous fat (%)	0.9±0.1	1.4±0.1*	0.9±0.2	1.8±0.4*
Epididymal fat (%)	1.3±0.2	1.5±0.2	1.4±0.1	1.9±0.3
Mesenteric fat (%)	0.6±0.0	0.5±0.0*	0.6±0.1	0.9±0.1
Retroperitoneal fat (%)	1.2±0.1	1.4±0.2	2.9±0.4	6.7±0.6*

Data are expressed as means±SEM (n = 4–5 per group). Fat pad weights are expressed as a percentage of fasted body weight. *p<0.05 vs controls.

**Figure 1:**
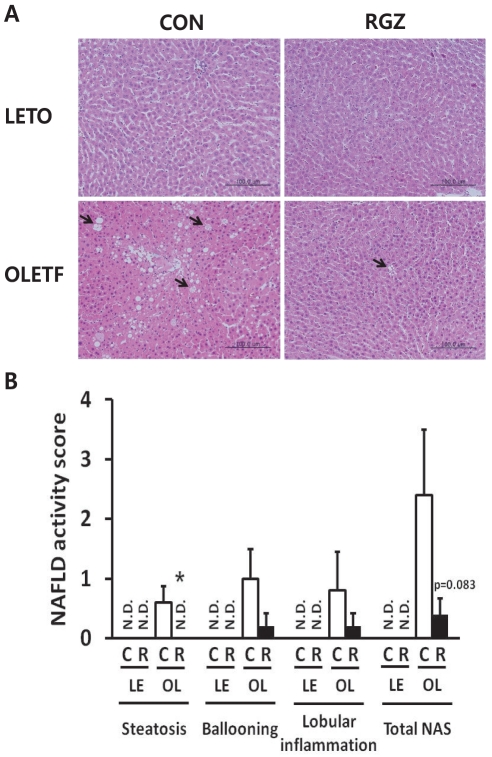
Rosiglitazone (RGZ) improves hepatic steatosis and nonalcoholic fatty liver disease (NAFLD) in OLETF rats. (A) Representative photographs of hematoxylin and eosin-stained sections of livers. Arrows indicate macrovesicular- and microvesicular steatosis. Scale bar, 100 µm. Original magnification, x200 for light microscopy. (B) NAFLD activity score (NAS). N.D., not detected; LE, LETO; OL, OLETF; R, RGZ; Data for NAS are means±SEM (n = 4–5 per group). *p<0.05 vs controls (CON or C).

**Figure 2:**
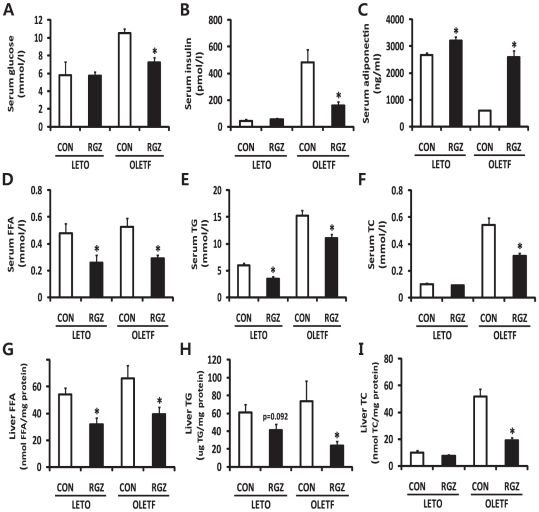
The effects of rosiglitazone (RGZ) on serum and liver metabolic parameters. Values of liver free fatty acids (FFA), triglycerides (TG), and total cholesterol (TC) were normalized with respect to protein concentrations. Data are means±SEM (*n* = 4–5 per group). *p<0.05 vs controls (CON).

Histological analyses demonstrated that OLETF livers were filled with large lipid droplets and there was evidence of both macrovesicular- and microvesicular steatosis, while LETO livers showed normal morphology (Figure 1A). RGZ-treated OLETF livers showed a significant reduction in the degree of steatosis (p = 0.040), and a trend of decrease in NAFLD activity score compared with untreated OLETF rats (p = 0.083; Figure 1B). In addition, serum levels of glucose and insulin were reduced by RGZ treatment in OLETF rats (Figures 2A, B). RGZ treatment increased serum adiponectin concentrations in both LETO and OLETF rats (Figure 2C). Levels of FFA, TG, and TC in serum and livers were decreased in RGZ-treated OLETF rats compared with untreated rats (Figures 2D–I). Reduced lipid droplets and TG in RGZ-treated OLETF livers confirmed that RGZ improves hepatic steatosis [13].

### RGZ Up-regulated Gene Expression of Sirt6 and Increased phosphorylation levels of LKB1 and AMPK in OLETF Rat Livers

**Figure 3:**
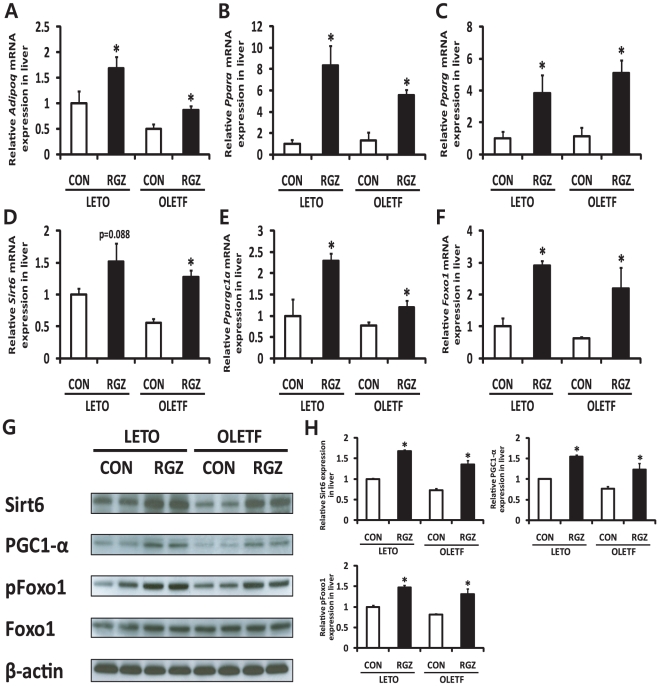
Rosiglitazone (RGZ) alters the expression of Sirt6 and other related targets in rat livers. Gene expression of (A) adiponectin (*Adipoq*), (B) peroxisome proliferator-activated receptor alpha (*Ppara*), (C) PPAR gamma (*Pparg*), (D) Sirt6, (E) peroxisome proliferator-activated receptor gamma coactivtor-1-α (*Ppargc1a*), and (F) forkhead box O1 (*Foxo1*). The expression levels were normalized with respect to those of glyceraldehyde-3-phosphate dehydrogenase (*Gapdh*). (G) Representative western blots for Sirt6, peroxisome proliferator-activated receptor gamma coactivtor-1-α (PGC1- α), phosphorylated Foxo1 at Ser249 (pFoxo1), Foxo1, and β-actin. (H) Densitometric analysis of Sirt6, PGC1- α, and pFoxo1. The expression of Sirt6 and PGC1- α was normalized to values for β-actin. Levels of phosphorylated Foxo1 at Ser249 were normalized to values for Foxo1. Data are means±SEM (*n* = 4–5 per group). *p<0.05 vs controls (CON).

**Figure 4:**
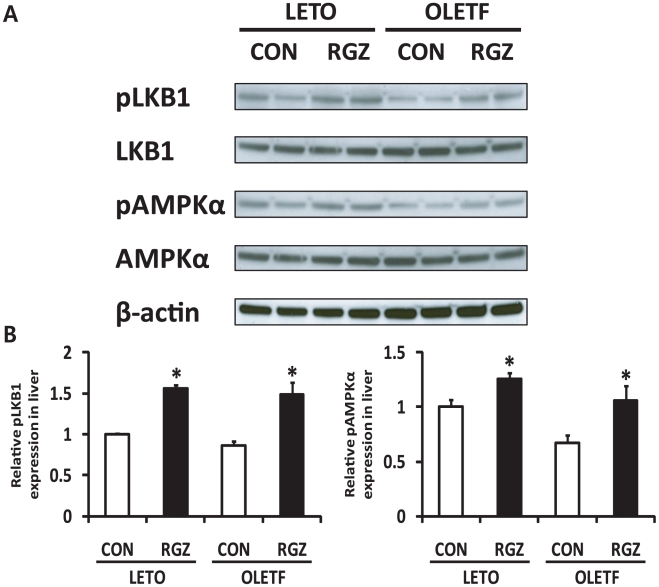
Rosiglitazone (RGZ) activates the LKB1-AMP-activated protein kinase (AMPK) pathway in rat livers. (A) Representative western blots for phosphorylated LKB1 at Ser428 (pLKB1), LKB1, phosphorylated AMPKα at Thr172 (pAMPKα), pAMPKα, and β-actin. (B) Densitometric analysis of pLKB1 and pAMPKα. Levels of phosphorylated LKB1 at Ser428 and AMPKα at Thr172 were normalized to values for LKB1 and AMPKα, respectively. Data are means±SEM (n = 4–5 per group). *p<0.05 vs controls (CON).

Next, we explored the involvement of Sirt6 and AMPK in the functional regulation of RGZ by investigating whether RGZ alters expression of Sirt6 and other related genes in the liver. Gene expression of adiponectin (*Adipoq*), PPAR alpha (*Ppara*), and *Pparg* was significantly increased in both LETO and OLETF rat livers with RGZ (Figures 3A–C). Moreover, RGZ treatment significantly up-regulated expression of Sirt6, Ppargc1a/PGC1-α, and Foxo1 in OLETF rat livers (Figures 3D–H). AMPK plays a key role in the regulation of lipid metabolism by controlling fatty acid oxidation and synthesis, and its activation is reported to be affected by Sirt1 [23], [24]. The phosphorylation levels of AMPK at Thr172 and LKB1 at Ser428 were significantly increased by RGZ treatment (Figure 4), suggesting that the Sirt6-AMPK pathway may be involved in the beneficial effects of RGZ on hepatic steatosis.

### RGZ-Dependent Reduction of Lipid Accumulation and Activation of the Sirt6-AMPK Pathway Is Abrogated by Knockdown of Sirt6 in AML12 Mouse Hepatocytes

**Figure 5:**
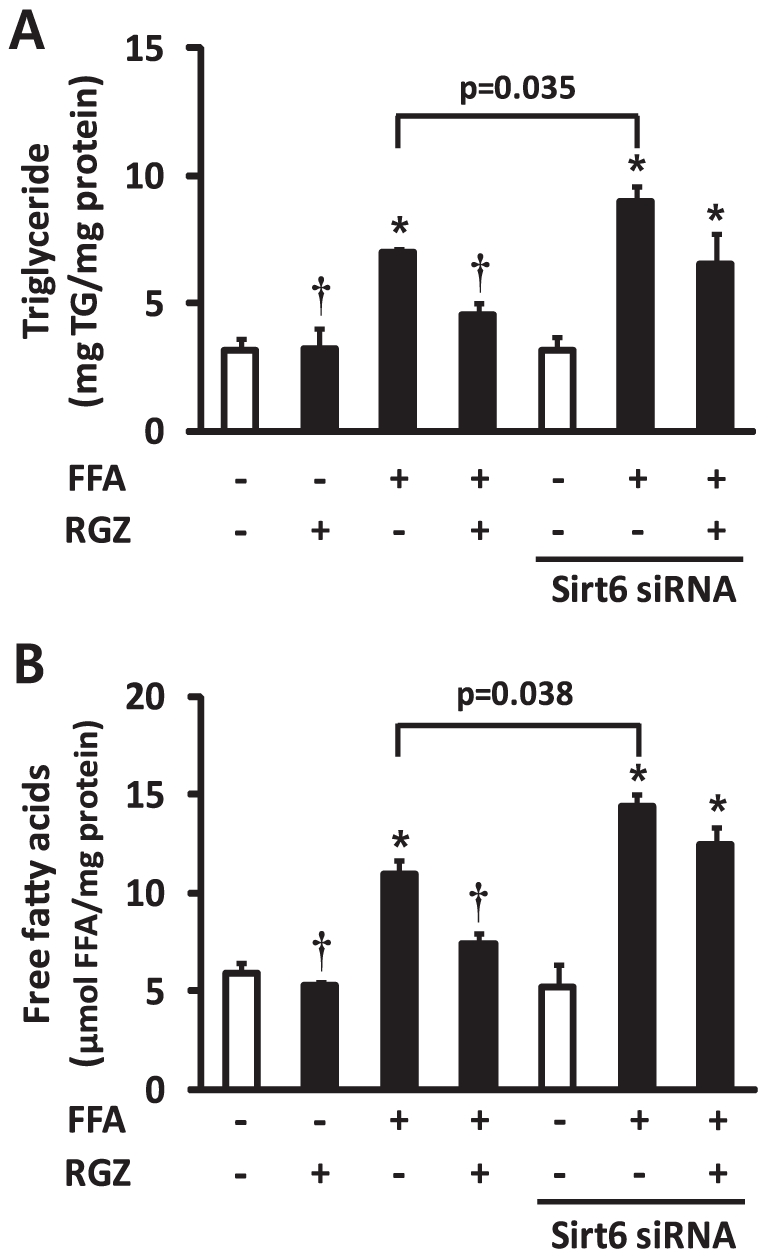
Sirt6 knockdown diminished the effect of rosiglitazone (RGZ) to reduce hepatocyte lipid accumulation. Values of triglyceride (TG) and free fatty acids (FFA) were normalized with respect to protein concentrations. Data are means±SEM (*n* = 6 per group). *p<0.05 vs control (CON; white bar), †p<0.05 vs FFA alone.

**Figure 6:**
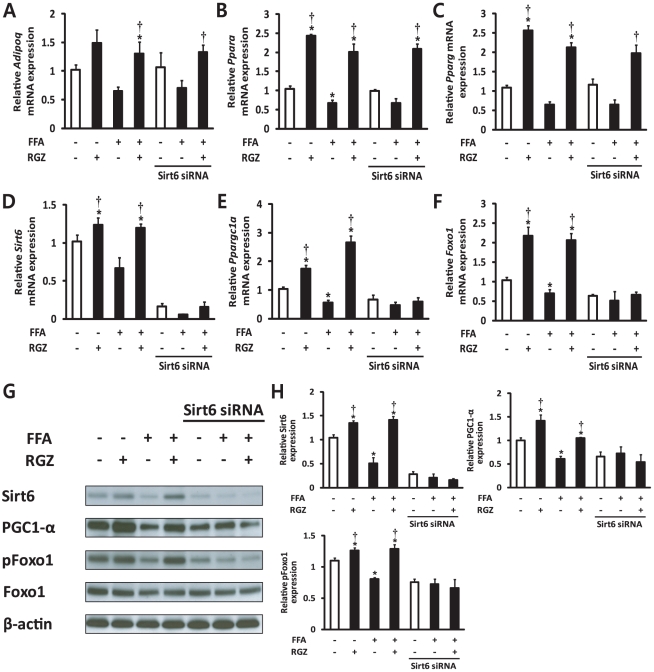
The effect of Sirt6 knockdown on expression of Sirt6 and related targets in hepatocytes. Gene expression of (A) adiponectin (*AdipoQ*), (B) peroxisome proliferator-activated receptor alpha (*Ppara*), (C) PPAR gamma (*Pparg*), (D) Sirt6, (E) peroxisome proliferator-activated receptor gamma coactivtor-1-α (*Ppargc1a*), and (F) forkhead box O1 (*Foxo1*). Expression levels were normalized with respect to those of β-actin (*Actb*). (G) Representative western blots for Sirt6, peroxisome proliferator-activated receptor gamma coactivtor-1-α (PGC1- α), phosphorylated Foxo1 at Ser249 (pFoxo1), Foxo1, and β-actin are shown. (H) Densitometric analysis of Sirt6, PGC1- α, and pFoxo1. The expression of Sirt6 and PGC1- α was normalized to values for β-actin. Levels of phosphorylated Foxo1 at Ser249 were normalized to values for Foxo1. FFA, free fatty acids; RGZ, rosiglitazone. Data are means±SEM (*n* = 6 per group). *p<0.05 vs control (CON; white bar), †p<0.05 vs FFA alone.

The *in vivo* experiments suggested that coordinated regulation of adiponectin, Sirt6, and AMPK may play a role in RGZ's protective action. The up-regulation of Sirt6 and its related genes as well as the activation of LKB1 and AMPK by RGZ led us to hypothesize that RGZ may exert its positive effects by acting on Sirt6. We therefore performed RNAi-mediated gene silencing by transfecting AML12 cells with siRNA oligos targeting Sirt6. Subsets of cells were incubated with FFA for 48 h to induce hepatocyte steatosis, and then treated with FFA and/or RGZ for additional 24 h. FFA incubation for 72 h induced hepatocyte steatosis with a significant increase in TG and FFA (Figure 5). As expected, RGZ treatment significantly reduced hepatocyte lipid accumulation. However, Sirt6 knockdown significantly increased TG and FFA levels in FFA-treated hepatocytes and diminished the effects of RGZ on hepatocyte lipid accumulation (Figure 5). The mRNA and protein expression patterns of targets that were investigated were similar to those found *in vivo* (Figure 6). Sirt6 knockdown was confirmed by a significant repression of its mRNA and protein expression (Figures 6D, G, H). Gene expression of *Adipoq*, *Ppara*, and *Pparg* was up-regulated by RGZ and the up-regulation was not altered with Sirt6 knockdown, suggesting that these may act as upstream regulators of Sirt6 (Figures 6A–C). However, the RGZ-mediated up-regulation of Ppargc1a/PGC1-α and Foxo1 was abolished with Sirt6 knockdown, suggesting that Ppargc1a/PGC1-α and Foxo1 are downstream targets of Sirt6 in AML12 mouse hepatocytes (Figures 6E–H). Sirt6 knockdown has no effect on Sirt1 mRNA expression level (control siRNA, 1.07±0.31; Sirt6 siRNA, 1.11±0.21 relative fold change; p = 0.888).

Mediators involved in insulin signaling were analyzed to see whether Sirt6 knockdown in AML12 mouse hepatocytes affects the insulin signaling pathway. As expected, RGZ increased protein expression of GLUT1 and phosphorylation levels of IR at three tyrosine residues (Tyr1158, Tyr1162, and Tyr1163), IRS1 at Tyr612, and Akt at Ser473 in the presence or absence of FFA (Figure S1). Sirt6 knockdown increased protein expression of GLUT1 and phosphorylation levels of IRS1 at Tyr612 and Akt at Ser473 (Figure S1).

**Figure 7:**
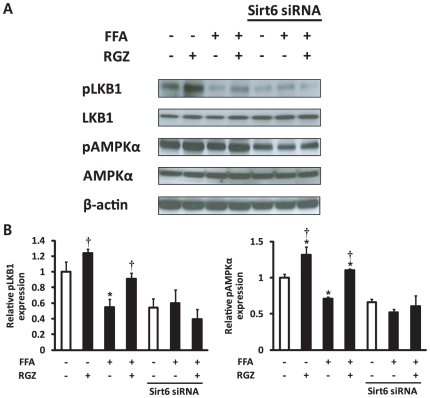
The effect of Sirt6 knockdown on phosphorylation of LKB1 and AMP-activated protein kinase (AMPK) in hepatocytes. (A) Representative western blots for phosphorylated LKB1 at Ser428 (pLKB1), LKB1, phosphorylated AMPKα at Thr172 (pAMPKα), pAMPKα, and β-actin. (B) Densitometric analysis of pLKB1 and pAMPKα. Levels of phosphorylated LKB1 at Ser428 and AMPKα at Thr172 were normalized to values for LKB1 and AMPKα, respectively. FFA, free fatty acids; RGZ, rosiglitazone. Data are means±SEM (n = 6 per group). *p<0.05 vs control (CON; white bar), †p<0.05 vs FFA alone.

Lastly, we investigated whether the phosphorylation levels of LKB1 and AMPK is altered by Sirt6 knockdown with RGZ treatment in AML12 mouse hepatocytes. RGZ increased phosphorylation levels of LKB1 (Ser428) and AMPK (Thr172) in the presence of FFA, and the knockdown of Sirt6 suppressed the effects of RGZ on the phosphorylation of LKB1 and AMPK (Figure 7), suggesting that Sirt6 regulates the phosphorylation of LKB1 and AMPK.

## Discussion

In this paper, we have demonstrated that RGZ improved hepatic steatosis, accompanied by an elevation in adiponectin, Sirt6 and its related targets, as well as AMPK phosphorylation, both *in vivo* and *in vitro*. The protective effects of RGZ and other TZDs against hepatic steatosis were previously reported in patients with type 2 diabetes and nonalcoholic steatohepatitis [11], [12], [28]. Similarly, improvements in hepatic steatosis and oxidative stress have been observed in rats with high fructose-induced metabolic syndrome [13]. Thus, the beneficial effect of rosiglitazone against hepatic steatosis was supported by a number of findings, although the underlying functional mechanisms remained to be established.

It is well known that TZD regulates insulin action, lipid and glucose metabolism and energy homeostasis by impacting PPARγ and its transcriptional regulation [6], [7]. RGZ significantly increases the release of adiponectin, presumably acting through PPARγ [17] which activates AMPK, leading to stimulation of glucose utilization and fatty acid oxidation [29]. In addition to its PPARγ-dependent action, TZD has been suggested to also control metabolic processes via PPARγ-independent mechanisms. RGZ is known to increase AMPK activation by inhibition of complex I activity of the respiratory chain, which results in an increase in the cellular AMP:ATP ratio [16], [30]. Here, we found that RGZ treatment increased adiponectin, PPARα, PPARγ and AMPK activation, suggesting that these factors may be involved in the RGZ-mediated amelioration of hepatic steatosis. RGZ likely acts through the PPARγ-induced increase in adiponectin and the subsequent elevation of AMPK activity as well as PPARα via adiponectin receptor 1 (Adipor1) and Adipor2, respectively. A recent paper demonstrated that adenovirus-mediated expression of Adipor1 and Adipor2 stimulated AMPK activation and PPARα signaling, respectively, resulting in increased fatty acid oxidation and reduced TG in the livers of *db/db* mice [31].

We also observed up-regulation of Sirt6 and its potential downstream targets, Ppargc1a and Foxo1, with RGZ treatment, *in vivo* and *in vitro*. From these observations, we hypothesize that Sirt6 may be a key regulating target for RGZ to reduce hepatic fat accumulation. To determine whether Sirt6 is causally related to the development of hepatic steatosis and its amelioration by RGZ, we directly inhibited Sirt6 by RNAi-mediated gene silencing in AML12 mouse hepatocytes. The knockdown of Sirt6 aggravated hepatocyte fat accumulation as shown by increased TG levels, and suppressed the favorable effects of RGZ on hepatocyte steatosis. In addition, Sirt6 knockdown suppressed gene expression of Ppargc1a and Foxo1, and abolished the RGZ-mediated activation of LBK1 and AMPK, suggesting that Ppargc1a, Foxo1, LKB1 and AMPK may act as down-stream effectors of Sirt6. These data are similar to previous findings for Sirt1 and LKB1/AMPK signaling. Sirt1 is known to act as upstream of LKB1/AMPK signaling and LKB1 is required for AMPK activation by Sirt1 in HepG2 cells and in the mouse liver [23]. Sirt1 regulation of AMPK signaling may be an indirect effect of Sirt1-mediated deacetylation of LKB1, and its subsequent translocation to the cytoplasm where it is activated [24]. Sirt1 also deacetylates other targets, such as Ppargc1a and Foxo1 [32], [33], and this deacetylation and subsequent activation enables these mediators to participate in Sirt1-AMPK-dependent metabolic regulation. Given our findings that Sirt6 knockdown suppressed activation of LKB1 and AMPK, we hypothesize that Sirt6 also acts as upstream of AMPK signaling. However, our data do not exclude the possibility that the AMPK signaling pathway also controls the expression and activity of Sirt6. In mouse skeletal muscles and C2C12 skeletal muscle cells, AMPK increases cellular NAD^+^ levels, leading to elevated Sirt1 activity [34], [35]. The increased Sirt1 activity results in deacetylation and modulation of activities of Sirt1 downstream targets. These findings support a role for AMPK also in stimulating the activities of Sirt1 and Sirt6 via an increase in cellular NAD^+^ levels. Considering that AMPK regulation on Sirt1 has up to date been exclusively reported in skeletal muscles, it is possible that the interdependence of Sirt1/6 and AMPK is tissue-specific. The relationship between Sirt1/6 and AMPK is more likely dominated by Sirt1/6 regulation on LKB1/AMPK signaling, especially in the liver.

Over the past several years, the role of sirtuins has expanded to that of metabolic regulators, in addition to their function as NAD^+^-dependent deacetylases. Overexpression of Sirt1 and Sirt6 was recently reported to improve glucose control, hepatic steatosis and inflammation [22], [26]. Conversely, liver-specific Sirt1 knockout mice developed hepatic steatosis, hepatic inflammation, and endoplasmic reticulum stress [21]. Similar aggravation of hepatic steatosis was also observed in liver-specific Sirt6 knockout mice [36]. And, the Sirt6 knockout mice showed severe hypoglycemia by enhancing insulin signaling and subsequent glucose uptake [37]. Similarly, mediators involved in insulin signaling and glucose uptake were increased by Sirt6 knockdown in AML12 mouse hepatocytes. Treatment of HepG2 cells and mice with Sirt1 activators (e.g., resveratrol and SRT1720) showed therapeutic potential for the treatment of fatty liver [38], [39]. Like other Sirt1 activators, RGZ administration increased Sirt6 expression and phosphorylation levels of LKB1 and AMPK, and provided protection from hepatic steatosis, suggesting that RGZ may act as a Sirt6 activator and a therapeutic strategy for NAFLD. Regarding the involvement of Sirt1 in the RGZ's action, a recent paper using a mouse model of alcoholic fatty liver showed that RGZ improves alcoholic fatty liver and stimulates adiponectin-Sirt1-AMPK signaling [40]. Moreover, in rat hepatoma cells, adiponectin activated AMPK signaling by up-regulating Sirt1 [40]. These findings were the first to demonstrate the involvement of Sirt1 in the protective action of RGZ against alcoholic fatty liver. The regulatory effects of adiponectin, Sirt1, and AMPK found in rat hepatoma cells are intriguing, but, future studies are needed to investigate whether this regulation is RGZ-dependent. In light of previous findings, our data demonstrating the involvement of Sirt6 in the RGZ-mediated protection of hepatic steatosis suggest that Sirt6 may have functional similarities with Sirt1 as a metabolic regulator. Both Sirt1 and Sirt6 are essential for survival as demonstrated by knockout of Sirt1 or Sirt6 that resulted in postnatal death in mice [25], [41]. Moreover, both are located in the nucleus [18], implying their possible involvement in transcriptional regulation of genes modulating metabolic processes. Sirt1 and Sirt6 also both show protective effects against hepatic steatosis [22], [26]. Considering the previous observations as well as our findings, it can be speculated that both Sirt1 and Sirt6 are involved in the RGZ-mediated amelioration of hepatic steatosis. However, how and what extents the interaction between Sirt1 and Sirt6 affects and which sirtuin contributes more to the RGZ's action is not yet completely known. A provocative observation was reported that Sirt1 is involved in the transcriptional regulation of Sirt6 by forming a complex with Foxo3a and nuclear respiratory factor 1 on the Sirt6 promoter [36]. However, Sirt6 knockdown had no regulatory effect on Sirt1 mRNA expression in AML12 mouse hepatocytes. Additional studies are required to investigate how Sirt1 or Sirt6 interacts each other or with other sirtuins, and to determine how the involvement of specific types of sirtuins in the metabolic processes is regulated. In addition, more studies are needed to investigate metabolic regulation by Sirt6 and its underlying mechanisms.

In this study, we aimed to elucidate whether Sirt6 is involved in RGZ-mediated protection against hepatic steatosis. Consistent with the previous reports, our findings provide evidence that RGZ improves hepatic steatosis. Interestingly, we found that the RGZ-mediated beneficial effect against hepatic steatosis is dependent on the Sirt6-AMPK pathway. Our study is the first to demonstrate the involvement and importance of Sirt6 in RGZ-mediated protection against hepatic steatosis. The metabolic effects of RGZ are mediated by the induction of a genetic network controlling fatty acid oxidation. Regulation occurs by multiple mechanisms that involve the direct deacetylation of Ppargc1a/PGC1-α, Foxo1, and other targets, as well as the indirect stimulation of AMPK signaling. Our findings emphasize that Sirt6 is involved in RGZ-mediated metabolic regulation. Taken together, these findings support the improvement of hepatic steatosis by RGZ by activation of the Sirt6-AMPK pathway, and suggest Sirt6 as a therapeutic target for hepatic steatosis.

## Supporting Information

Figure S1 **The effect of Sirt6 knockdown on mediators involved in insulin signaling and glucose uptake in hepatocytes.** Representative western blots for phosphorylated insulin receptor (IR) at Tyr1158, Tyr1162, and Tyr1163 (pIR(Tyr)), IR, phosphorylated IR substrate1 (IRS1) at Tyr612, IRS1, phosphorylated Akt at Ser473, Akt, glucose transporter 1 (GLUT1), and β-actin. FFA, free fatty acids; RGZ, rosiglitazone.
(EPS)
